# ANGPTL4: A Predictive Marker for Diabetic Nephropathy

**DOI:** 10.1155/2019/4943191

**Published:** 2019-10-27

**Authors:** Eman Al Shawaf, Mohamed Abu-Farha, Sriraman Devarajan, Zahra Alsairafi, Irina Al-Khairi, Preethi Cherian, Hamad Ali, Aditi Mathur, Fahd Al-Mulla, Abdulnabi Al Attar, Jehad Abubaker

**Affiliations:** ^1^Biochemistry and Molecular Biology, Dasman Diabetes Institute, Kuwait; ^2^National Dasman Diabetes Biobank, Dasman Diabetes Institute, Kuwait; ^3^Department of Pharmacy Practice, Faculty of Pharmacy, Kuwait; ^4^Functional Genomic Unit, Dasman Diabetes Institute, Kuwait; ^5^Department of Medical Laboratory Sciences, Faculty of Allied Health Sciences, Health Sciences Center, Kuwait University, Kuwait; ^6^Diabetology Unit, Amiri Hospital, Dasman Diabetes Institute, Kuwait

## Abstract

**Background:**

ANGPTL4 is a glycoprotein that is involved in regulating triglyceride metabolism by inhibiting LPL activity under fasting conditions. Additionally, ANGPTL4 has been suggested as a link between hypertriglyceridemia and albuminuria in the nephrotic syndrome. In this study, we examined levels of circulating ANGPTL4 in people with diabetic nephropathy (DN) and its association with established DN-associated proteins such as IGFBP1 and IGFBP4.

**Methods:**

We quantified circulating ANGPTL4, IGFBP1, IGFBP3, and IGFBP4 in fasting plasma samples of 122 Kuwaiti participants using a multiplexing assay. The study involved 36 controls, as well as 86 people with type 2 diabetes (T2D) including 37 people with normal kidney function and 49 people with DN.

**Results:**

ANGPTL4 level was increased in people with DN (241.56 ± 14.1 *μ*g/ml) compared to the control group (178.43 ± 24.09 *μ*g/ml). The increase in ANGPTL4 correlated with clinical parameters of DN including albumin-to-creatinine ratio (*r* = 0.271, *P* = 0.002), serum creatinine (*r* = 0.381, *P* = 0.0001), and eGFR (*r* = −0.349, *P* < 0.0001). Furthermore, ANGPTL4 correlated positively with both IGFBP1 (*r* = 0.202, *P* = 0.026) and IGFBP4 (*r* = 0.364, *P* < 0.0001). Multiple regression analysis showed increased IGFBP1 and TG as predictors of higher ANGPTL4 in people with DN. In people with T2D, only IGFBP1 acted as a positive predictor of a rise in ANGPTL4.

**Conclusion:**

In this study, our data showed a significant increase in circulating ANGPTL4, IGFBP1, and IGFBP4 in patients with DN. The elevation in ANGPTL4 correlated significantly with clinical markers of DN such as ACR, serum creatinine, and eGFR, as well as IGFBP1 and IGFBP4. Altogether, this suggests a potential role for ANGPTL4 in DN perhaps through its role in inhibiting LPL activity and promotes ANGPTL4 as a biochemical marker for the detection of a diabetic kidney disease in patients with T2D.

## 1. Introduction

Type 2 diabetes is a multifactorial disease, characterized by dysfunctional pancreatic activity and insulin resistance (IR). Many studies have associated IR with moderate and severe chronic renal failure, and the presence of IR was recently shown at the early stages of mild renal disease in people with diabetic nephropathy [1, 2]. The current clinical practice uses the estimated glomerular filtration rate (eGFR) and urinary albumin-to-creatinine ratio (ACR) as the standard methods for assessing glomerular damage and changes to renal function. However, the precision and reliability of these indicators are affected by various factors, making them less accurate for an early detection of a diabetic kidney disease [3]. Additional biochemical markers that would aid in the detection and understanding of the pathophysiology of DN to minimise its deleterious consequences are highly valuable.

Angiopoietin-like protein 4 (ANGPTL4) is a glycoprotein (≈45-65 kDa) secreted by a wide range of cells such as adipocytes, hepatocytes, myocytes, macrophages, and endothelial and intestinal cells [4]. Since its discovery, ANGPTL4 has been extensively investigated, reporting its involvement in a myriad of physiological and pathological conditions including energy homeostasis, tumorigenesis, angiogenesis, wound healing, and redox regulation [5]. One of the extensively investigated roles of ANGPTL4 is its role in lipid metabolism, specifically as a potent inhibitor of lipoprotein lipase (LPL) that would regulate LPL activity to suppress the clearance of triglycerides (TG) from the circulation. In this regard, loss of ANGPTL4 expression would reduce circulating TG, whereas its overexpression elevates TG levels [5]. Through this regulatory mechanism, ANGPTL4 would help in protecting cells from lipotoxicity by reducing extracellular TG hydrolysis and the consequent fatty acid uptake [5]. On the other hand, deficiencies affecting LPL activity would consequently lead to hypertriglyceridemia, which is a key feature of nephrotic syndrome. In the work of Clement et al. [6], ANGPTL4 was reported as the missing link between hypertriglyceridemia and albuminuria in the nephrotic syndrome [7].

Development of the diabetic kidney syndrome involves various biological factors including members of the insulin-like growth factor-binding proteins (IGFBPs). This family of proteins comprises six high-affinity binding proteins (IGFBP1–IGFBP6) that modulate the bioactivity of insulin-like growth factor (IGF) ligands, IGF1 and IFG2. The circulating hepatic protein, IGFBP1, has been implicated in a number of physiological processes including metabolic homeostasis, glucose counter regulation [8], insulin sensitivity [9], and cardiovascular pathophysiology [10, 11]. At a state of diabetic kidney syndrome, renal IGFBP1 was found to increase at both mRNA and protein levels [12]. Albeit the involvement of IGFBP3 in diabetic microalbuminuria and DN has been studied [12], little is known about the role of IGFBP1 in DN. Overexpression of IGFBP1 was suggested to affect glomerulosclerosis [13], and an increased hepatic production of IGFBP1 was linked to a decline in IGF1 serum levels in people with diabetes [12].

In this study, we wanted to explore a potential correlation between IGFBP1 and ANGPTL4 under conditions of T2D alone and DN, where a rise in ANGPTL4 levels would be permissive for the development of a kidney condition as a complication of diabetes. This would promote the use of ANGPTL4 as a biochemical marker for the detection of nephropathy in patients with diabetes.

## 2. Materials and Methods

### 2.1. Study Population

The study involved three main groups that were matched by body mass index and age: individuals with T2D but without DN (T2D group, *n* = 37), people with T2D and DN (DN group, *n* = 49), and people with no T2D and no nephropathy (control group, *n* = 36), as described previously. All participants were recruited at Dasman Diabetes Institute (Dasman, Kuwait). Written consents were obtained from all participants prior to their enrollment in the study. This study was approved by the Ethical Review Board of Dasman Diabetes Institute (DDI) as abiding by the ethical guidelines outlined in the Declaration of Helsinki.

### 2.2. Anthropometric and Biochemical Measurements

Fasting blood samples were collected in Vacutainer-EDTA tubes and centrifuged for 10 minutes at 400 × g for plasma extraction. Collected plasma samples were aliquoted and stored at -80°C until assayed. Blood pressure values are the average of three consecutive readings and were measured with an Omron HEM-907XL digital sphygmomanometer. Fasting blood glucose (FBG), serum total cholesterol (TC), low-density lipoprotein (LDL-C), high-density lipoprotein (HDL-C), and triglycerides (TG) were measured by a Siemens Dimension RxL chemical analyzer (Diamond Diagnostics, Holliston, MA, USA). Levels of albumin and creatinine were quantified in urine samples using the CLINITEK Novus Automated Urine Chemical Analyzer (Siemens Healthineers, Erlangen, Germany).

### 2.3. Creatinine and Urinary Protein Detection

Urinary protein was quantified using a Coomassie Plus protein assay kit following the manufacturer's protocol (Pierce, Rockford, IL, USA). Creatinine levels, both urinary and serum, were measured by a VITROS 250 automatic analyzer (NY, USA). The estimated glomerular filtration rate (eGFR) was calculated using the Modification of Diet in Renal Disease study equation.

### 2.4. ANGPTL4 Enzyme-Linked Immunosorbent Assay (ELISA)

ANGPTL4 protein plasma levels were quantified using a Magnetic Luminex Assay kit (R&D Systems Europe, Ltd., Abingdon, UK) following the manufacturer's protocol. Plasma samples were thawed on ice, while repeated freeze-thaw cycles were avoided. Cross reactivity with other proteins was not significant.

### 2.5. IGFBP1, IGFBP3, and IGFBP4 Assay

Levels of IGFBP1, IGFBP3, and IGFBP4 were determined by the Magnetic Luminex Assay kit (R&D Systems Europe, Ltd., Abingdon, UK) following the manufacturer's protocol.

### 2.6. Statistical Analysis

One-way analysis of variance (ANOVA) was used to compare diabetes, diabetes with nephropathy, and control data with Bonferroni post hoc tests for multiple comparisons to determine statistical significance. The correlation between ANGPTL4 and various parameters was estimated by the Spearman correlation coefficient. The stepwise multiple regression model was employed to identify the parameters independently associated with ANGPTL4. All data are presented as mean ± SEM, with a *P* value < 0.05 indicating significance. All statistical analyses were performed using GraphPad Prism software (La Jolla, CA, USA) and SPSS for Windows version 25.0 (IBM SPSS Inc., USA).

## 3. Results

This study involved 122 Kuwaiti participants that were classified into three groups: control with no diabetes or nephropathy (*n* = 36), people with diabetes (*n* = 37), and people with diabetes and nephropathy (*n* = 49). Participants were diagnosed with nephropathy by having an elevated urine albumin-to-creatinine ratio (ACR) > 30 mg/g. A detailed description of the study population's demographic, clinical, and biochemical characteristics is presented in Table 1. Participants from all groups were age and BMI matched with no significant statistical difference between the groups (*P* > 0.05). In Table 1, our data shows that participants with DN had a significant increase in TG, FBG, HbA1c, and serum creatinine levels (*P* < 0.05) compared to other groups.

### 3.1. Levels of Plasma ANGPTL4 and IGFBP Proteins Are Elevated with Nephropathy

**Figure 1:**
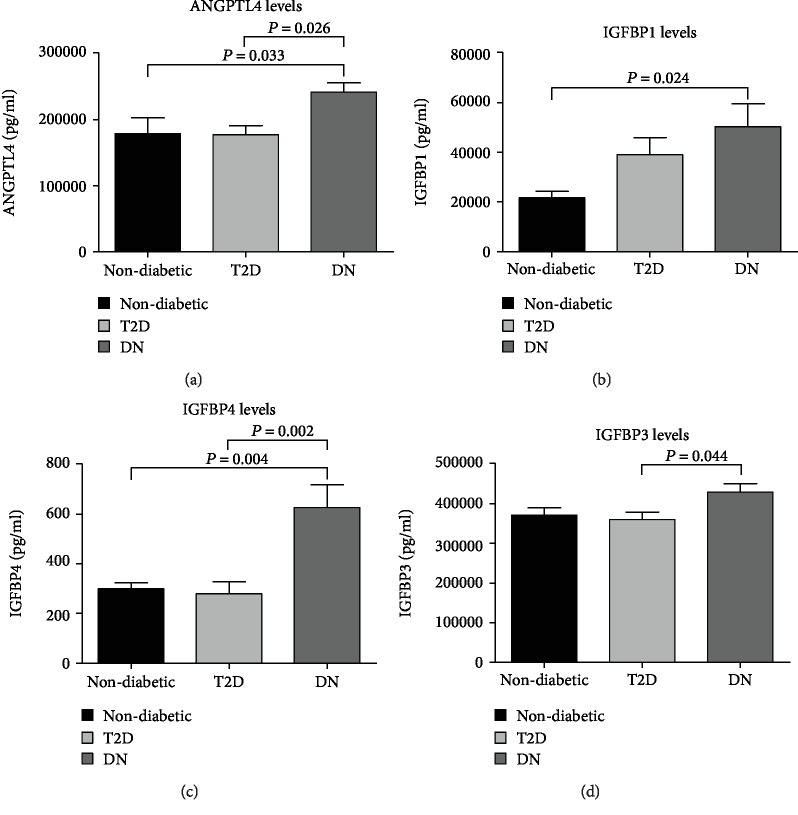
Fasting plasma levels of ANGPTL4, IGFBP1, IGFBP4, and IGFBP3 in all populations. (a) Comparing levels of ANGPTL4 between all study groups: nondiabetic control, T2D, and DN. (b) Comparing levels of IGFBP1 between control, T2D, and DN patients. (c) Comparing IGFBP4 levels between control, T2D, and DN patients. (d) Comparing IGFBP3 levels between control, T2D, and DN patients.

Levels of circulating ANGPTL4 (Figure 1(a)) were significantly higher in patients with DN (241.56 ± 14.2 *μ*g/ml) compared to those with T2D (176.88 ± 14.11 *μ*g/ml, *P* = 0.026) and control (178.43 ± 2409 *μ*g/ml, *P* = 0.033). Measurement of IGFBPs showed that levels of circulating IGFBP1 were significantly higher in patients with DN compared to the control group (21.6 ± 2.67 *μ*g/ml, *P* = 0.024, Figure 1(b)). However, the difference in IGFBP1 level was not significant comparing people with DN (50.27 ± 9.34 *μ*g/ml) to people with T2D (39.02 ± 7.04 *μ*g/ml). Circulating IGFBP4 was significantly higher in patients with DN (0.63 ± 0.09 *μ*g/ml) compared to patients with T2D (0.28 ± 0.05 *μ*g/ml, *P* = 0.002) and control (0.29 ± 0.03 *μ*g/ml, *P* = 0.004, Figure 1(c)). Levels of circulating IGFBP3 were almost comparable between the study groups (Figure 1(d)); however, IGFBP3 was significantly higher in patients with DN (429.51 ± 21.36 *μ*g/ml, *P* = 0.044) compared to people with T2D (360.2 ± 18.69 *μ*g/ml). There was no significant difference in levels of IGFBP3 between people from the control group (371.46 ± 18.48 *μ*g/ml) and people with T2D and DN.

### 3.2. ANGPTL4 Is Correlated with Clinical Parameters and IGFBPs

**Figure 2:**
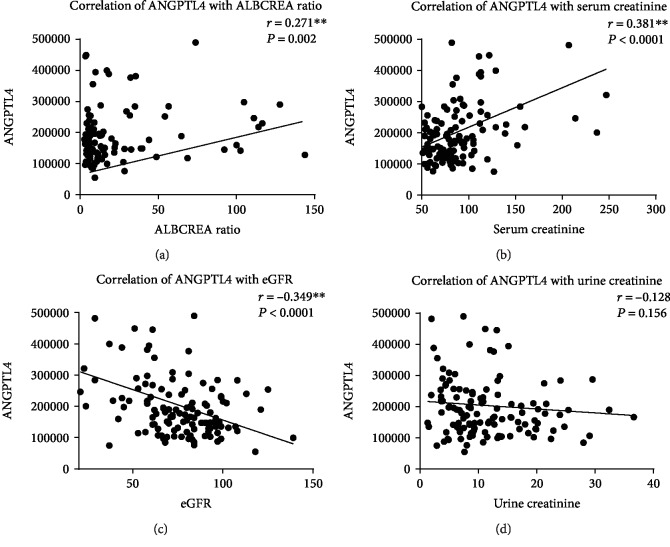
Correlation analysis between ANGPTL4 and clinical parameters associated with DN. Correlation analysis between ANGPTL4 and (a) ACR, (b) serum creatinine, (c) eGFR, and (d) urine creatinine.

**Figure 3:**
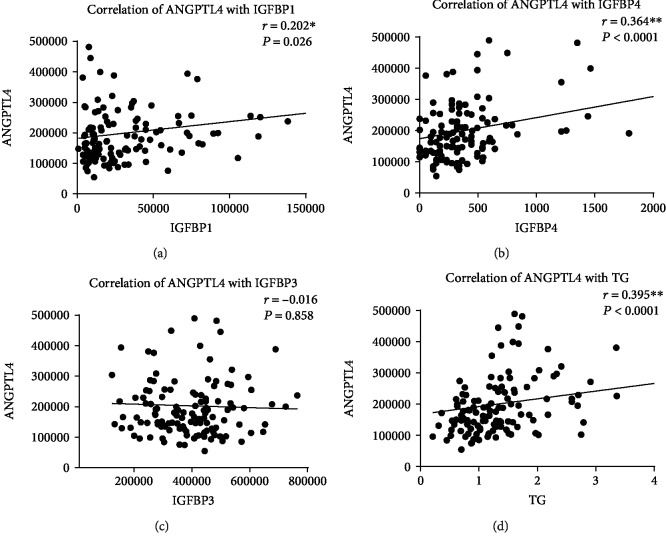
Correlation analysis between ANGPTL4, IGFBPs, and TG. Correlation analysis between ANGPTL4 and (a) IGFBP1, (b) IGFBP4, (c) IGFBP3, and (d) TG.

To evaluate the relationship between ANGPTL4 and clinical parameters in diabetes and nephropathy, a correlation analysis was conducted. According to Spearman's coefficient, ANGPTL4 was positively correlated with both albumin-to-creatinine ratio (*r* = 0.271, *P* = 0.002) and serum creatinine (*r* = 0.381, *P* = 0.0001, Figures 2(a) and 2(b), respectively). The correlation between ANGPTL4 and a state of DN was further confirmed through a negative correlation between ANGPTL4 and eGFR (*r* = −0.349, *P* < 0.0001, Figure 2(c)). We have also looked at the correlation between ANGPTL4 and DN (i.e., defined by having abnormal ACR levels) in a group of DN participants with normal TG levels. Our correlation analysis shows that in people with normal TG levels, there is a significant correlation (*r* = 0.355, *P* = 0.018) between ANGPTL4 and DN. The correlation between ANGPTL4 and urine creatinine was not significant (Figure 2(d)). Investigating the relationship between ANGPTL4 and circulating IGFBPs, we found a positive correlation between ANGPTL4 and two IGFBPs (Figures 3(a) and 3(b)), IGFBP1 (*r* = 0.202, *P* = 0.026) and IGFBP4 (*r* = 0.364, *P* < 0.0001) in our total study population. To scrutinize these correlations, we examined it in individual study groups finding a significant positive correlation between ANGPTL4 and IGFBP1 in both people with T2D (*r* = 0.411, *P* = 0.011) and people with DN (*r* = 0.471, *P* = 0.003). On the other hand, the correlation between ANGPTL4 and IGFBP4 was only significant in people with T2D (*r* = 0.341, *P* = 0.039). In our analysis, we found no significant correlation between ANGPTL4 and IGFBP3 (Figure 3(c)). In our data, ANGPTL4 was positively correlated with TG (*r* = 0.395, *P* < 0.0001, Figure 3(d)), which appeared to be specific to people with DN (*r* = 0.462, *P* = 0.001).

### 3.3. Multiple Regression Analysis to Predict ANGPTL4 Elevation in Nephropathy

To further examine the association between ANGPTL4 and clinical markers, we employed a stepwise multiple regression model with a number of predictors (Table 2). In the group with T2D, both HDL-C and IGFBP1 were used as predictors, and this produced *R*^2^ = 0.43 and *F*(2, 33) = 7.90 (*P* < 0.001). In people with T2D, IGFBP1 appeared to be a marker with significant positive regression weight (*β* = 0.382, *P* = 0.012). In participants with DN, the regression model produced *R*^2^ = 0.34 and *F*(2, 43) = 10.96 (*P* < 0.001) with TG and IGFBP1 as predictors of an increase in the circulating levels of ANGPTL4. Both TG and IGFBP1 markers had significant positive regression weights (*β* = 0.417, *P* = 0.002 and *β* = 0.451, *P* = 0.001, respectively). Taken altogether, at a condition of diabetes both with and without nephropathy, IGFBP1 was found as a marker and a significant predictor with the dependent variable ANGPTL4.

Stepwise multiple regression model analysis was performed to identify parameters associated with ANGPTL4 in our study groups (T2D, DN, and healthy control). The analysis revealed that IGFBP1 is independently associated with ANGPTL4 in both T2D (*β* = 0.382, *P* = 0.012) and DN (*β* = 0.451, *P* = 0.001) groups. Moreover, the circulating HDL-C (*β* = −0.584, *P* < 0.001) and TG (*β* = 0.417, *P* = 0.002) were predictors of ANGPTL4 in T2D and DN study groups, respectively. Taken altogether, at a condition of diabetes both with and without nephropathy, IGFBP1 was found to be a marker and a significant predictor with the dependent variable ANGPTL4.

## 4. Discussion

In this study, we showed that circulating ANGPTL4 was significantly increased in patients with DN and it was significantly correlated with clinical markers of DN. IGFBP1 and IGFBP4 were also elevated in people with DN and showed a significant positive correlation with ANGPTL4. Using a multiple regression model, we showed that the increased levels of IGFBP1 and TG are strong predictors of a rise in circulating ANGPTL4, which consequently indicates a state of nephropathy in people with diabetes.

Diabetic nephropathy is one of the most common microvascular complications of diabetes; it is a progressive condition influenced by various biological factors and characterized by a gradual deterioration of the kidney function. This begins with glomerular hyperfiltration, thickening of the basement membrane, and mesangial expansion leading to glomerular sclerosis and tubular interstitial fibrosis [14]. Early detection of a diabetic kidney disease is crucial to better clinical management and to avoiding reaching the end-stage renal disease. Typically, the diabetic kidney disease is characterized by a persistent albuminuria and elevated serum creatinine with a progressive decline in eGFR [15]. Nonetheless, staging of DN is governed by various markers, some of which are only effective at late stages of the disease such as eGFR (detecting CKD ≥ stage 3). Other markers like albuminuria is a dynamic, fluctuating condition that does not follow a linear progressive process [15]. Nonetheless, the presence of albuminuria is broadly accepted as an indicative of advanced renal structural changes in the kidney reflecting an established state of nephropathy [16, 17]. For this reason, identifying novel biochemical markers for the detection of DN is crucial.

ANGPTL4 plays a key role in lipid metabolism and has been linked to a range of physiological and pathophysiological conditions. Studies investigating ANGPTL4 in T2D and nephropathy are limited. However, in agreement with our finding, a study involving children with the minimal change disease, which is a common cause of the nephrotic syndrome, reported an overproduction of ANGPTL4 in the glomerulus (i.e., the filtering unit of the kidney) [6]. Chugh et al. also found the pathogenesis of the nephrotic syndrome to involve an upregulation of ANGPTL4 expression in podocytes and the development of proteinuria [18]. Overexpression of ANGPTL4 was shown to induce a nephrotic-range proteinuria in rats [6]. In our study, we report a significant increase in circulating ANGPTL4 level in patients with DN compared to people with T2D. Interestingly, levels of ANGPTL4 in people with T2D and control were comparable. Thus, ANGPTL4 in both groups was lower compared to that in people with DN (Figure 1(a)). Additionally, clinical markers of DN like ACR and serum creatinine were positively correlated with ANGPTL4, while glomerular filtration rate reflected by eGFR was negatively correlated with ANGTPL4. Collectively, our correlation analysis (Figure 2) displayed a clear link between the rise in circulating ANGPTL4 and kidney disease. In our study population, elevated levels of ANGPTL4 were restricted to a condition of nephropathy and it was significantly correlated with clinical markers of DN, suggesting ANGPTL4 as a parameter that would help in characterizing a condition of the diabetic nephrotic syndrome.

The pathogenesis of DN involves an interplay between various proteins, ligands, and cellular subtypes. Considering this fact, we investigated the involvement of other potential proteins in DN with a possible correlation with ANGPTL4. Components of the GH-IGF-IGFBP axis have a crucial role in both retaining a normally functional renal system and the development of DN [12]. IGF1 is one of the main factors involved in the development of DN; IGFBPs which regulate the bioavailability and actions of IGF1 appear to contribute to the pathology of a diabetic kidney. In our study, we found people with DN to have a significant increase in their plasma IGFBP1 and IGFBP4 (Figures 1(b) and 1(c)). The rise in IGFBP1 was reported previously in an animal model of the diabetic kidney disease. This study found an association between increased levels of renal IGFBP1 protein and renal IGF1 level [12, 19]. Furthermore, levels of both IGFBP1 and IGFBP4 were detected in the glomerular ultrafiltrate of diabetic rats [12, 20]. Our data shows an increase in circulating IGFBP1 in people with diabetes, both with and without nephropathy (Figure 1(b)). However, circulating IGFBP4 was only significantly high in people with DN (Figure 1(c)). To explain the rise of IGFBP1 in people with diabetes, previous studies investigating the role of IGFBP1 found it as a good predictive marker for abnormal glucose homeostasis [21, 22]. Additionally, IGFBP1 was found to acutely regulate glucose levels by affecting free IGF1, thus playing a role in glucose metabolism [23]. Moreover, increasing levels of circulating IGFBP1 have been linked to hyperglycemia [24], and overexpression of IGFBP1 in diabetes was shown to contribute to glomerulosclerosis [12]. As for IGFBP4, in a previous study, its serum level was fourfold higher in children with chronic renal failure, and this increase was related to the degree of renal dysfunction [25]. Our study is limited by the cross-sectional design that should be considered upon data interpretation. Thus, future studies are required to elucidate the mechanism through which ANGPTL4 and other potential molecules are contributing to a state of DN. It would also be interesting to measure levels of ANGPTL4 in nondiabetic kidney disease patients, to understand if the ANGPTL4 elevation is specific to DN or is a feature of any kidney disease. Collectively, our data showed people with DN to have elevated levels of IGFBP1 and IGFBP4, which comes in agreement with previous reports and suggests the elevation of these IGFBPs as indicative factors of a diabetic kidney disease.

In this study, we report a positive correlation between ANGPTL4 and IGFBP1, IGFBP4, and TG in the general population (Figure 3). Increased ANGPTL4 levels correlated positively with IGFBP1 in people with diabetes with and without nephropathy. Nonetheless, the correlation between ANGPTL4 and IGFBP4 was specific to people with T2D. The significance of these correlations was emphasized through a multiple regression model to predict changes in ANGPTL4 levels. Our data suggest ANGPTL4 as a strong positive predictor in people with DN, with both IGFBP1 and TG showing a significant positive regression weight. In people with T2D, IGFBP1 showed a significant positive regression weight, while HDL-C showed a negative correlation. In this regard, the multiple regression model indicates that increased levels of IGFBP1 and TG are strong predictors of a rise in circulating ANGPTL4, which consequently indicates a state of nephropathy in people with diabetes.

In the current clinical practice, markers that are used to predict the onset and progression of DN have prognostic limitations. This raised the need to look for alternative biomarkers, which would facilitate the identification of patients at an increased risk of DN. Our data suggests ANGPTL4 as a significant positive marker linked to DN. Nonetheless, further investigation is required to have a better understanding of the way ANGTPL4 contributes to the nephrotic syndrome.

## 5. Conclusion

To conclude, our data showed a significant increase in levels of circulating ANGPTL4, which was exclusive to people with DN. Interestingly, the presence or absence of T2D did not have a significant effect on circulating ANGPTL4. Additionally, ANGPTL4 was positively correlated with IGFBP1 and IGFBP4, which were linked to DN in previous reports. Thus, our data suggest ANGPTL4 as a novel biomarker that can be employed as a predictive marker for a nephrotic syndrome risk.

## Figures and Tables

**Table 1:** Anthropometric and clinical characteristics of participants with diabetes and diabetes and nephropathy and healthy controls.

Parameters	Nondiabetic controls (*N* = 36)	T2D (*N* = 37)	T2D with DN (*N* = 49)	*P* value
Age (years)	55.03 ± 1.17	57.27 ± 1.11	59.00 ± 1.32	0.077
Gender M/F	15/21	16/21	34/15	0.013
Body mass index (kg/m^2^)	30.16 ± 0.69	31.56 ± 0.60	31.88 ± 0.64	0.150
SBP (mmHg)	121.64 ± 2.29	131.6 ± 2.46	130.98 ± 5.36	0.200
DBP (mmHg)	73.56 ± 1.56	72.46 ± 1.78	68.51 ± 3.01	0.282
Fasting glucose (mmol/l)	5.45 ± 0.11	7.86 ± 0.41	8.67 ± 0.44	≤0.001
HbA1C (%)	5.66 ± 0.09	7.59 ± 0.19	7.87 ± 0.25	≤0.001
TC (mmol/l)	4.85 ± 0.16	4.10 ± 0.16	3.87 ± 0.12	≤0.001
TG (mmol/l)	1.09 ± 0.09	1.39 ± 0.20	1.70 ± 0.13	0.016
HDL-C (mmol/l)	1.46 ± 0.06	1.21 ± 0.6	1.10 ± 0.04	≤0.001
LDL-C (mmol/l)	3.72 ± 0.85	2.28 ± 0.13	2.01 ± 0.11	0.020
VLDL (mmol/l)	0.44 ± 0.04	0.56 ± 0.08	0.68 ± 0.05	0.016
C-peptide (pg/ml)	0.71 ± 0.05	0.67 ± 0.05	0.66 ± 0.06	0.829
Serum creatinine (mg/l)	76.31 ± 3.09	73.08 ± 3.14	109.57 ± 6.28	≤0.001
eGFR (ml/min/1.73 m^2^)	80.22 ± 2.27	86.64 ± 3.03	64.08 ± 3.48	≤0.001
Albumin (mcg/l)	40.50 ± 0.58	39.11 ± 0.50	37.51 ± 0.53	0.001
CRP (*μ*g/ml)	0.32 ± 0.07	0.38 ± 0.07	0.46 ± 0.09	0.489
ACR (mg/g)	9.99 ± 1.34	10.24 ± 1.12	707.07 ± 217.78	0.001
Urine creatinine (mg/day)	14.47 ± 1.35	12.62 ± 1.07	8.78 ± 0.84	0.001
Microalbumin (mg/day)	14.97 ± 2.24	13.57 ± 1.56	592.87 ± 226.96	0.010

Data are mean ± standard error mean. SBP: systolic blood pressure; DBP: diastolic blood pressure; ACR: albumin/creatinine ratio. ^∗∗^*P* < 0.05 with nondiabetic controls; ^∗^*P* < 0.05 with nondiabetic controls.

**Table 2:** Multiple regression analysis to identify parameters associated with ANGPTL4 in nondiabetic controls, T2D, and T2D with DN.

Parameters	Nondiabetic controls		T2D		T2D with DN	
	*β*	*P* value	*β*	*P* value	*β*	*P* value
HDL-C	-**0.346**	**0.039**	-**0.584**	<**0.001**	-0.104	0.483
TG	-0.228	0.243	0.037	0.799	**0.417**	**0.002**
IGFBP1	-0.112	0.499	**0.382**	**0.012**	**0.451**	**0.001**

Dependent variable ANGPTL4.

## Data Availability

Data will only be shared upon request from the corresponding author due to unpublished data and ethical restriction by the institute.

## Abbreviations

T2D: Type 2 diabetes mellitus
DN: Diabetic nephropathy
BMI: Body mass index
SBP: Systolic blood pressure
DBP: Diastolic blood pressure
HDL-C: High-density lipoprotein cholesterol
LDL-C: Low-density lipoprotein cholesterol
TC: Total cholesterol
TG: Triglycerides
hsCRP: High-sensitivity C-reactive protein
FBG: Fasting blood glucose
HbA1c: Hemoglobin A1c
ANGPTL4: Angiopoietin-like protein 4
eGFR: Glomerular filtration rate
ACR: Albumin-to-creatinine ratio
IR: Insulin resistance
LPL: Lipoprotein lipase
IGFBPs: Insulin-like growth factor-binding proteins
IGF: Insulin-like growth factor.
